# Alleles of HLA-DRB1*04 Associated with Pulmonary Tuberculosis in Amazon Brazilian Population

**DOI:** 10.1371/journal.pone.0147543

**Published:** 2016-02-22

**Authors:** Dhêmerson Souza de Lima, Mauricio Morishi Ogusku, Maisa Porto dos Santos, Cláudia Maria de Melo Silva, Vanessa Alves de Almeida, Irineide Assumpção Antunes, Antonio Luiz Boechat, Rajendranath Ramasawmy, Aya Sadahiro

**Affiliations:** 1 Programa de Pós-Graduação em Imunologia Básica e Aplicada, Instituto de Ciências Biológicas, Universidade Federal do Amazonas (UFAM), Manaus, Amazonas, Brasil; 2 Laboratório de Micobacteriologia, Instituto Nacional de Pesquisas da Amazônia (INPA), Manaus, Amazonas, Brasil; 3 Programa de Pós-Graduação em Ciências Farmacêuticas, Faculdade de Ciências Farmacêuticas (FCF), Manaus, Amazonas, Brasil; 4 Policlínica Cardoso Fontes, Manaus, Amazonas, Brasil; 5 Fundação de Medicina Tropical Doutor Heitor Vieira Dourado (FMT/HVD), Manaus, Amazonas, Brasil; 6 Universidade Nilton Lins, Manaus, Amazonas, Brasil; Charite Universitätsmedizin Berlin, GERMANY

## Abstract

Immunogenetic host factors are associated with susceptibility or protection to tuberculosis (TB). Strong associations of HLA class II genes with TB are reported. We analyzed the HLA-DRB1*04 alleles to identify subtypes associated with pulmonary TB and their interaction with risk factors such as alcohol, smoking, and gender in 316 pulmonary TB patients and 306 healthy individuals from the Brazilian Amazon. The HLA-DRB1*04 was prevalent in patients with pulmonary TB (*p*<0.0001; OR = 2.94; 95% CI = 2.12 to 4.08). Direct nucleotide sequencing of DRB1 exon 2 identified nine subtypes of HLA-DRB1*04. The subtype HLA-DRB1*04:*11*:*01* (*p* = 0.0019; OR = 2.23; 95% CI = 1.34 to 3.70) was associated with susceptibility to pulmonary TB while DRB1*04:*07*:*01* (*p*<0.0001; OR = 0.02; 95% CI = 0.001 to 0.33) to protection. Notably, the interaction between alcohol and HLA-DRB1*04:*11*:*01* increased the risk for developing pulmonary TB (*p* = 0.0001; OR = 51.3; 95% CI = 6.81 to 386). Multibacillary pulmonary TB, the clinical presentation of disease transmission, was strongly associated with interaction to alcohol (*p* = 0.0026; OR = 11.1; 95% CI = 3.99 to 30.9), HLA-DRB1*04:*11*:*01* (*p* = 0.0442; OR = 2.01; 95% CI = 1.03 to 3.93) and DRB1*04:*92* (*p* = 0.0112; OR = 8.62; 95% CI = 1.63 to 45.5). These results show that HLA-DRB1*04 are associated with pulmonary TB. Interestingly, three subtypes, DRB1*04:*07*:*01*, DRB1*04:*11*:*01* and DRB1*04:*92* of the HLA-DRB1*04 could be potential immunogenetic markers that may help to explain mechanisms involved in disease development.

## Introduction

Tuberculosis (TB), a serious public health problem worldwide, is caused by *Mycobacterium tuberculosis* complex (MTBC) with *M*. *tuberculosis* being the most common [1, 2]. According to the World Health Organization (WHO), approximately one-third of the world’s population is infected with TB Bacillus and two billion people are estimated to have latent TB infection with risk for development of the disease. Of notified TB cases, more than 90% occur in low and middle-income countries [3]. Brazil reported 67.966 TB cases in 2014, an incidence rate of 33.5 cases of TB/100.000 habitants. In 2014, the Amazonas state had an incidence rate of 68.4 cases of TB/100.000 habitants that is well above the national average and ranks first in relation to other states [4].

Several risk factors such as HIV-infected individuals [5, 6], diabetes mellitus [7, 8], smoking [9], alcohol [10, 11], under-nutrition [12, 13] and host immunogenetic factors [14–18] are associated with susceptibility to TB. Host genetic factors are strongly associated with the development of TB as hardly 5% to 10% of MTB-infected individuals develop the disease [19, 20]. Several studies have associated alleles of the Human Leukocyte Antigen (HLA) class II to TB [21–29]. Particularly, the following alleles HLA-DRB1*0803, DRB1*0601, DRB1*04, DRB1*09, DRB1*10, DRB1*15 and DRB1*16 are shown to be associated with susceptibility to pulmonary TB [28, 30–33]. This diversity of alleles is certainly related to the high polymorphism of the HLA system [34–36]. A limitation of these studies is the use of techniques of low-resolution typing that not identified the allele subtypes.

In preliminary studies, the generic HLA-DRB1*04 was frequent in pulmonary TB patients (unpublished data), but this gene has many subtypes and is very important to determine which alleles are associated with the disease.

For this reason, our study aimed to identifying subtypes of HLA-DRB1*04 in patients with pulmonary TB to correlate with risk factors for the development of disease and to explain the HLA role on the high incidence rate of TB cases in the Amazonas state.

## Materials and Methods

### Population Samples

A total of 622 individuals, aged 18 to 60 years, born in the Brazilian Amazon are non-indigenous and their parents and grandparents were also born and lived in the same region. All of the patients with Pulmonary TB (n = 316) participating in this study are unrelated, treatment-naïve, positive for sputum smear or culture tests and were selected at the Reference Center for Sanitary Pneumology, Policlínica Cardoso Fontes, Amazonas, Manaus, Brazil. Patients with treatment abandonment or recurrence, autoimmune diseases, cancer, diabetes, HIV, or using immunoregulatory drugs were excluded. Pregnant women were also excluded. The control group (n = 306) consisted of direct contacts of patients recently diagnosed with pulmonary TB, and were without signs and symptoms of the disease and negative for bacteriological tests.

### Mycobacteriology

Bacteriological tests were performed at the Micobacteriology Laboratory, of Instituto Nacional de Pesquisas da Amazônia (INPA). The sputum samples were processed for the realization of direct or concentrated sputum smear and culture by PKO method [37, 38]. Patients were classified as multibacillary (individuals who had positive direct sputum smear or concentrated) and paucibacillary (individuals with negative sputum smear, but with positive results for the culture method).

### DNA extraction and PCR of the HLA-DRB1*04 allele

Genomic DNA was extracted from peripheral blood leucocytes by using the rapid technique tetramethylammonium bromide salts (DTAB/CTAB) [39] and stored at -20°C for use in PCR. The following pair of primers: Forward 5’ GT TTC TTG GAG CAG GTT AAA C 3’ and Reverse 5’ CCT AAA CCT TCA CCC CAA CCA C 3’ was used for the amplification of the specific allele for HLA-DRB1*04. The PCR optimization was performed. Each reaction contains a mix of 1X Buffer (20 mM Tris-HCl pH 8.4, 50 mM KCl), 0.2 mM of each dNTPs, 2.0 mM MgCl_2_, 0.2 uM of forward and reverse primers, 1.25 U of *Taq* DNA polymerase (Invitrogen) and 4 uL of genomic DNA (50 ng/uL), in a total volume of 25 uL. The PCR program was an initial denaturation at 96°C for 5 minutes, followed by 35 cycles at 96°C for 1 minute, 64.5°C for 1 minute, 72°C for 1 minute, and a final extension at 72°C for 10 minutes in a Veriti Thermal Cycler. PCR products were detected in a 1.5% agarose gel electrophoresis stained with SYBR Green and visualized in blue-light transilluminator. The electrophoresis was run in TBE buffer for 60 minutes at 130 V and 110 mA.

### Purification of the PCR product and nucleotide sequencing of the HLA-DRB1*04

PCR products were purified using polyethylene glycol (PEG) 20%. The sequencing reaction was performed by the dideoxynucleotides method [40] using the BigDye Terminator Kit version 3.1 to sequence the fragments in both directions (forward and reverse) in the automated sequencer ABI 3130xl Genetic Analyzer (Applied Biosystems). The consensus sequences were built with Geneious software version R8 and compared with the database sequences IMGT/HLA (BLASTn) (http://www.ebi.ac.uk/ipd/imgt/hla/).

### *In silico* prediction of HLA class II molecules binding ESAT-6

The predictions binding between early secreted antigenic target of 6 kDa (ESAT-6) of *Mycobacterium tuberculosis* H37Rv (accession number Rv3875) with HLA class II molecules were carried out by bioinformatics tools using analysis of algorithms ("*silico* mapping") by the method of Artificial Neural Network (NetMHCIIpan)[41] in the Immune Epitope Database on and Analysis Resource (IEDB) (http://www.iedb.org/home_v3.php). Lower percentile rank indicates higher affinity (Cut-off percentile rank ≤ 10.0).

### Ethics

This study was approved by the Human Research Ethics Committee of the Universidade Federal do Amazonas (reference number: 0017.0.115.000–08) according to the Brazilian Federal laws. All study participants provided written informed consent.

### Statistical analysis

Descriptive statistic was used to characterize the profile of pulmonary TB patients and controls involved in this study. For the comparison of HLA frequencies in patients and controls, 2x2 contingency tables analysis was performed using Chi-square or Fisher's Exact test. The level of significance was *p*<0.05 and 95% confidence interval. Stepwise logistic regression was performed with *p*<0.05 and confidence interval of 95% to assess the general characteristics of patient and control groups. Interaction analysis of HLA-DRB1*04 gene with the general characteristics and bacterial load (multibacillary and paucibacillary) were carried out by stepwise logistic regression. The precision and accuracy (goodness of fit) of each stepwise logistic regression model were analyzed with the area under the ROC curve (AUC≥0.7) and the Hosmer-Lemeshow test (*p*>0.05). A formal Bonferroni correction for the number of HLA-DRB1*04 analysed would require significance threshold of *p*<0.006 (p0/n, p0 = 0.05, n = 9 HLA-DRB1*04 subtypes). Statistics analysis were performed in GraphPad Prism version 6 and MedCalc version 15 softwares.

## Results

### Baseline characteristics of the study population

Of the 316 patients with pulmonary TB, 75% were classified as multibacillary and 25% as paucibacillary. Baseline characteristics of patients and controls are shown in Table 1. There were no significant differences in mean age and BCG-vaccination. There were, however, significant differences between genders. Pulmonary TB was more common in males. Alcohol and smoking were associated with the disease.

**Table 1:** General characteristics of patients with pulmonary TB and controls.

Characteristics	Patients n = 316	Controls n = 306
**Gender**^a^		
Male, n (%)	194 (61.4%)	142 (46.4%)
Female, n (%)	122 (38.6%)	164 (53.6%)
Mean age ± SD	36±12.6	35±10.7
**BCG-vaccinated**		
Yes, n (%)	212 (67.0%)	180 (58.8%)
No, n (%)	25 (8.00%)	36 (11.8%)
Data not available, n (%)	79 (25.0%)	90 (29.4%)
**Alcoholic drink**^b^		
Yes, n (%)	87 (27.5%)	20 (6.50%)
No, n (%)	135 (42.7%)	226 (73.9%)
Data not available, n (%)	94 (29.8%)	60 (19.6%)
**Smoking**^c^		
Yes, n (%)	69 (21.8%)	9 (3.00%)
No, n (%)	153 (48.4%)	233 (76.0%)
Data not available, n (%)	94 (29.8)	64 (21.0%)

^a^ Gender: (*p* = 0.0002; OR = 1.84; 95% IC = 1.33 to 2.53), calculated with Fisher’s exact test

^b^ Alcoholic drink: Individuals who consumed ≥ 4 doses for women and ≥ 5 doses for men at the same time within the last 30 days [42]. 1 dose = 30 ml distilled (or 12 g of pure alcohol). *p*<0.0001; OR = 7.28; 95% CI = 4.28 to 12.4, calculated with Fisher’s exact test.

^c^ Smoking: were defined as smokers, who made use of cigarettes daily for one year or more [43]. *p*<0.0001; OR = 11.7; 95% CI = 5.66 to 24.1, calculated with Fisher’s exact test.

### PCR and nucleotide sequencing of the HLA-DRB1*04 allele

Of the 622 individuals participating in the study, 288 were positive for HLA-DRB1*04, 187 pulmonary TB patients (59.2%; 187/316) and 101 controls (33.0%; 101/306). HLA-DRB1*04 showed strong association with susceptibility to pulmonary TB (p<0.0001; OR = 2.94; 95% CI = 2.12 to 4.08). Nucleotide sequencing of the 288 HLA-DRB1*04 allele showed nine subtypes of HLA-DRB1*04 as listed in Table 2 when aligned with the sequences deposited in the database IMGT/HLA (BLASTn) (http://www.ebi.ac.uk/ipd/imgt/hla/), that also provides common alleles from different ethnicities.

**Table 2:** Results of the obtained nucleotide sequences and aligned with the sequences of the IMGT/HLA.

Alleles HLA-DRB1*04	Score	Length Obtained (IMGT)	Identities %	Positives %	Ethnic Origin IMGT/HLA
DRB1*04:*01*:*01*	1085	561 (801 bp)	100	100	Caucasoid, Oriental
DRB1*04:*02*:*01*	1085	561 (801 bp)	100	100	Caucasoid
DRB1*04:*03*:*01*	1085	561 (801 bp)	100	100	Caucasoid
DRB1*04:*04*:*01*	1085	561 (801 bp)	100	100	Caucasoid
DRB1*04:*05*:*01*	1085	561 (801 bp)	100	100	Caucasoid, Oriental
DRB1*04:*06*:*01*	1085	561 (801 bp)	100	100	Caucasoid, Oriental
DRB1*04:*07*:*01*	1076	561 (801 bp)	99	99	Caucasoid, Hispanic^a^
DRB1*04:*11*:*01*	1085	561 (801 bp)	100	100	Australian Aboriginal, H^a^, Mixed^b^
DRB1*04:*92*	1076	561 (663 bp)	99	99	Unknown ^c^

^a^ Hispanic: People historically of mixed Mediterranean Caucasoid, American Indian race

^b^ Mixed: The mixture of various races

^c^ Unknown: Ethnic origin unknown.

The distribution of the HLA-DRB1*04 allele subtypes in both pulmonary TB patients and controls is shown in Table 3. The most common subtypes in the study population were HLA-DRB1*04:*04*:*01* and HLA-DRB1*04:*11*:*01*. HLA-DRB1*04:*11*:*01* was strongly associated with susceptibility to pulmonary TB (*p* = 0.0019; OR = 2.23, 95% CI = 1.34 to 3.70). HLA-DRB1*04:*07*:*01* was associated with protection against the disease and was not found among the TB patients.

**Table 3:** Distribution and association analysis of HLA-DRB1*04 subtypes in pulmonary TB patients and the control group.

Alleles HLA-DRB1*04	Patientsn = 187 (%)	Controls n = 101 (%)	*p* value^a^	OR	95% CI
DRB1*04:*01*:*01*	6 (3.2)	6 (5.9)	0.3544	0.525	0.165 to 1.67
DRB1*04:*02*:*01*	9 (4.8)	9 (8.9)	0.2039	0.517	0.198 to 1.35
DRB1*04:*03*:*01*	9 (4.8)	9 (8.9)	0.2039	0.517	0.198 to 1.35
DRB1*04:*04*:*01*	42 (22.5)	28 (27.7)	0.3185	0.755	0.434 to 1.32
DRB1*04:*05*:*01*	2 (1.1)	3 (3.0)	0.3479	0.353	0.058 to 2.15
DRB1*04:*06*:*01*	3 (1.6)	2 (2.0)	1.0000	0.807	0.133 to 4.91
DRB1*04:*07*:*01*	0 (0.0)	12 (11.9)	0.0001^b^	0.019	0.001 to 0.33
DRB1*04:*11*:*01*	95 (50.8)	32 (31.7)	0.0019^b^	2.23	1.34 to 3.70
DRB1*04:*92*	21 (11.2)	0 (0.0)	0.0002^b^	26.21	1.57 to 438

^a^ Fisher’s exact test, *p* < 0.05; OR = Odds ratio; CI = Confidence interval

^b^ Significant p values according to the Bonferroni correction (*p*<0.006)

### The stepwise logistic regression models for HLA-DRB1*04 and pulmonary TB

Stepwise logistic regression analysis identified three risk factors. Alcohol (*p* = 0.0016; OR = 4.32; 95% CI = 1.74 to 10.7), male (*p* = 0.0001; OR = 2.69; 95% CI = 1.64 to 4.40) and smoking (*p* = 0.0442; OR = 3.16; 95% CI = 1.03 to 9.69) were associated with pulmonary TB.

To assess the influence of HLA-DRB1*04 gene on the risk factors, another stepwise logistic regression model was performed. HLA-DRB1*04 (*p* = 0.0143; OR = 1.92; 95% CI = 1.14 to 3.23) showed strong interaction with alcohol (*p*< 0.0001; OR = 8.43; 95% CI = 3.85 to 18.5) and increased the significant risk for development of pulmonary TB when compared to non-carriers of the allele. Nevertheless, HLA-DRB1*04 interaction to male (*p* = 0.0001; OR = 2.71; 95% CI = 1.66 to 4.43) or smoking (*p* = 0.0564; OR = 2.99; 95% CI = 0.97 to 9.26) did not affect significantly the risk of susceptibility to pulmonary TB.

Stepwise logistic regression model including the subtypes, HLA-DRB1*04:*11*:*01* and HLA-DRB1*04:*92* was carried out. Only the interaction of alcohol (*p* = 0.0001; OR = 51.3; 95% CI = 6.81 to 386) with HLA-DRB1*04:*11*:*01*(*p* = 0.0265; OR = 2.13; 95% CI = 1.09 to 4.14) showed a very high risk of susceptibility to pulmonary TB.

Stepwise logistic regression analysis on the effect of interaction of alcohol and HLA alleles on bacterial load (multibacillary and paucibacillary) showed that carriers of HLA-DRB1*04:*11*:*01* (*p* = 0.0442; OR = 2.01; 95% CI = 1.03 to 3.93) or HLA-DRB1*04:*92* (*p* = 0.0112; OR = 8.62; 95% CI = 1.63 to 45.5) who consume alcohol (*p* = 0.0026; OR = 11.1; 95% CI = 3.99 to 30.9) have higher risk of developing multibacillary pulmonary TB.

### *In silico* prediction of HLA class II molecules binding ESAT-6

NetMHCIIpan was used to estimate the binding affinity of ESAT-6 epitopes to HLA-DRB1*04:*11*:*01* and DRB1*04:*07*:*01*. Low binding affinity resulted for HLA-DRB1*04:*11*:*01* while strong binding for DRB1*04:*07*:*01*. The results are shown in percentile rank values (Table 4). Importantly, the core regions are fragments of antigens that interact with HLA molecules, determining binding affinity and specificity. Prediction analysis for HLA-DRB1*04:92 was not performed because this HLA is unavailable in IEDB database.

**Table 4:** Prediction of the binding for epitopes of ESAT-6 with HLA molecules encoded by HLA-DRB1*04:*11*:*01* and DRB1*04:*07*:*01*.

AllelesHLA-DRB1*04	Antigens_Start-End_	Sequences	Core regions	Percentile rank
DRB1*04:*07*:*01*	ESAT-6 _5–19_	QWNFAGIEAAASAIQ	FAGIEAAAS	1.58
DRB1*04:*07*:*01*	ESAT-6 _4–18_	QQWNFAGIEAAASAI	FAGIEAAAS	1.96
DRB1*04:*07*:*01*	ESAT-6 _6–20_	WNFAGIEAAASAIQG	FAGIEAAAS	3.78
DRB1*04:*07*:*01*	ESAT-6 _3–17_	EQQWNFAGIEAAASA	FAGIEAAAS	4.55
DRB1*04:*07*:*01*	ESAT-6 _7–21_	NFAGIEAAASAIQGN	FAGIEAAAS	8.31
DRB1*04:*07*:*01*	ESAT-6 _2–16_	TEQQWNFAGIEAAAS	FAGIEAAAS	9.92
DRB1*04:*11*:*01*	ESAT-6 _19–33_	QGNVTSIHSLLDEGK	VTSIHSLLD	13.28
DRB1*04:*11*:*01*	ESAT-6 _18–32_	IQGNVTSIHSLLDEG	VTSIHSLLD	13.54
DRB1*04:*11*:*01*	ESAT-6 _17–31_	AIQGNVTSIHSLLDE	VTSIHSLLD	16.08
DRB1*04:*11*:*01*	ESAT-6 _62–76_	ATELNNALQNLARTI	LNNALQNLA	16.22
DRB1*04:*11*:*01*	ESAT-6 _5–19_	QWNFAGIEAAASAIQ	FAGIEAAAS	16.68
DRB1*04:*11*:*01*	ESAT-6 _16–30_	SAIQGNVTSIHSLLD	VTSIHSLLD	16.71

Percentile rank ≤ 10.0—Lower percentile rank indicates the 15-mer peptides with higher affinity to HLA

### Frequency of HLA-DRB1*04 alleles in Worldwide Populations

Distribution of HLA-DRB1*04 were obtained in the database "Allele Frequency Database Net" (AFND) (http://www.allelefrequencies.net/). The HLA-DRB1*04 gene has the highest frequency in populations of the Americas (South = 42.0% and Central America = 33.9%). The subtype HLA-DRB1*04:*11*:*01* also is most common in the populations of the Americas (44.0%), although ethnic origin of this allele includes Australian aborigines, Hispanic and Mestizos (Table 2). The subtypes HLA-DRB1*04:*92* and HLA-DRB1*04:*07*:*01*, respectively, are more prevalent in the United States (0.0027%) and Mexico (34.5%) [44][44]. According to data provided, the subtypes associated with pulmonary TB (Table 3), are very frequent in the Americas.

## Discussion

In this study, the generic HLA-DRB1*04 allele was most common in pulmonary TB patients, showing strong association with susceptibility to disease. These data emphasize a major role of HLA system in the regulation of the immune response against pulmonary TB. A study conducted in Recife, Northeast Brazil, also identified HLA-DRB1*04 associated with pulmonary TB [45]. A recent meta-analysis of 31 studies with 3,416 TB patients and 4,515 controls also found in the Asian population HLA-DRB1*04 associated with susceptibility to TB [33]. Nevertheless, a study in Mexico found reduced risk for TB in carriers of HLA-DRB1*04 (OR = 0.24; 95% CI = 0.07 to 0.84) [46].

Most of the studies used only low-resolution HLA typing to search for association with TB. We performed nucleotide sequencing to look for which subtypes of the HLA-DRB1*04 allele are associated with susceptibility to TB. Interestingly, the subtype *HLA*-DRB1*04:*11*:*01* was strongly associated with TB in the Amazonas. Previous investigations reported other alleles subtypes within *DRB1* locus that were associated with risk to the disease among different populations such as the DRB1*15:*01* in south India [47], DRB1*08:*03* in Koreans [30], the same subtype and DRB1*08:01 in Kazaksthan with drug resistance [48]. The HLA alleles vary among populations because of genetic background. Of note, the HLA region is the most polymorphic in human genome [49].

In contrast to other studies, we also performed interaction of the susceptibility allele *HLA*-DRB1*04:*11*:*01* with alcohol consumption, smoking and gender. Importantly, there is the consumption of alcohol worldwide, with an average of 6.2 liters of pure alcohol per year. In Brazil the average alcohol consumption is 8.7 liters. The highest consumption levels are found in Europe and the Americas. Alcohol can lead to dependence and encourage the development of diseases [50]. Alcohol consumption contributes to development of pulmonary TB, favoring increased bacterial burden in lungs, besides causing a decrease in the number of CD4+ T cells and, consequently, lower release of IFN-y [51].

Recently, the meta-analysis of 72,684 individuals in 14 countries with high burden of tuberculosis, confirmed association with consumption of alcohol, smoking, and male gender [52]. Our study brings a further contribution to the meta-analysis study as for the first time, the interaction between risk factors of pulmonary TB disease development and HLA-DRB1*04 gene was analyzed. The combination of HLA-DRB1*04 gene and alcohol consumption increased twice as much the risk for pulmonary TB development. Surprisingly, the risk of TB considerably increased if there is the combination of HLA-DRB1*04:*11*:*01* subtype and alcohol consumption indicating the influence of genetic factor on susceptibility to TB disease. Nonetheless, the interaction of HLA-DRB1*04 gene to smoking, and gender did not influence the risk significantly. This observation is related to the reduced number of male’s smokers in the study. However, it is known that the population from the Northern region of Brazil has the lowest rate of smoking in the country, with 13.4% of smokers [42].

The strong association with HLA-DRB1*04:*11*:*01* subtype and susceptibility to TB, can be explained by the fact that this allele may possibly have low binding affinity as shown *in silico* prediction with epitopes of ESAT-6. ESAT-6 is an important protein released by TB bacillus. On the contrary, HLA-DRB1*04:*07*:*01*, associated with protection to TB disease, showed high binding affinity to six ESAT-6 epitopes as determined by the best binding [53]. Core regions of the epitopes (ESAT-6) are the fragments that interact with the molecule of HLA to determine the binding affinity and specificity [54]. The peptide residues immediately flanking the core regions contribute to HLA/peptide binding affinity [55].

These results support immunogenetic models, susceptibility and protection, and emphasize the importance the genetic background, in particular the HLA system that is essential for response of CD4+T cell with signaling by HLA class II/peptide, co-stimulation, and cytokines. Furthermore, good interaction between HLA/peptide have a crucial role for the development of a Th1 effector response against pathogen [56].Our data suggest that carriers of HLA-DRB1*04:*07*:*01* may have a good T cell response against TB bacillus while carriers of HLA-DRB1*04:*11*:*01* may have a poor immune response effector.

The HLA-DRB1*04 gene are associated with Rheumatoid arthritis (RA) [57–59]. However, the subtypes reported to be associated with RA are DRB1*0401, DRB1*0404, DRB1*0405, DRB1*0408, DRB1*0409 [60]. In our study, none of these subtypes are associated with TB. Interestingly, HLA-DRB1*04:*07*:*01* has been shown to increase the chance of developing RA [60] and this allele was associated with protection to TB in our study.

We also found HLA-DRB1*04:*11*:*01* and DRB1*04:*92*, were associated with multibacillary TB. Furthermore, there is a strong interaction of these *HLA* alleles with the alcohol consumption. These results suggest that multibacillary TB patients expressing one of these subtypes of *HLA* might be responsible for keeping the chain of disease transmission. Altogether, besides the risk factors associated with TB (socioeconomic, malnutrition, drug abuse, diabetes mellitus, HIV) [5, 61, 62], the genetic factor can also contribute to the maintenance of high rates of the disease in the Amazonas.

Different frequencies of *HLA* class II alleles are found worldwide, according to the geographic region. In South America HLA-DRB1*04 allele (generic) is more frequent in populations of Argentina and Brazil with a mean 39.6% and 22.0%, respectively [44]. HLA-DRB1*04:*11*:*01* subtype is also common in populations of Brazil and Argentina, with a mean frequency of 14.0% and 20.2%, respectively [44, 63]. Both HLA, are mostly found in the native population [44], and may indicate a rapid evolution of *HLA-DRB1* gene in ancestral Amerindians during migration processes [64]. HLA-DRB1*04:*92* is hardly found in the world and was associated with Black population of United States with an allele frequency of 0.0027% [44, 65]. HLA-DRB1*04:*07*:*01* has a mean frequency of 34.5% in the Mexican population [44, 66, 67]. It is highly probable that this is the allele associated with protection in Mexico since the generic HLA-DRB1*04 was associated with protection against pulmonary TB [46].

In conclusion, the present study showed HLA-DRB1*04 genes and their subtypes associated with pulmonary TB. Also, we determined that interaction between alcohol consumption and *HLA* gene increased the risk for the development of TB, and was associated with multibacillary TB clinical presentation. Further studies will be needed to confirm this association so as to start unraveling the complex network of responses in TB. The relationship between HLA and the immune response should be taken into consideration in future studies, specifically in the management of tuberculosis treatment.

## Limitations

The number of individuals with the HLA-DRB1*04:*07*:*01* or HLA-DRB1*04:*92* is small, therefore, require more research with larger numbers of patients with pulmonary TB and controls. Regarding the alcohol consumption and smoking, we did not assess the amount and frequency of consumption. Nevertheless, it is important to emphasize that the results of the statistical analysis were significant, with strong associations with pulmonary TB.

## Supporting Information

S1 Fig ROC curve for stepwise logistic regression model with alcoholic drink, smoking and gender (male) associated with pulmonary TB (AUC = 0.734). (TIF)

S2 Fig ROC curve of stepwise logistic regression model for variables alcoholic drink, gender (male) and their interaction to HLA-DRB1*04 gene, associated with pulmonary TB (AUC = 0.745). (TIF)

S3 Fig ROC curve of stepwise logistic regression model for alcoholic drink and their interaction to HLA-DRB1*04:*11*:*01*, associated with pulmonary TB (AUC = 0.732). (TIF)

S4 Fig ROC curve of stepwise logistic regression model for alcoholic drink, HLA-DRB1*04:*11*:*01* and HLA-DRB1*04:*92* associated with bacterial load (AUC = 0.743). (TIF)

S1 Table Stepwise logistic regression analysis with general characteristics of the population. (DOCX)

S2 Table Stepwise logistic regression for variables alcoholic drink, gender (male) and smoking, including the HLA-DRB1*04 gene. (DOCX)

S3 Table Stepwise logistic regression analysis for alcohol and HLA-DRB1*04:*11*:*01* allele associated with pulmonary TB. (DOCX)

S4 Table Stepwise logistic regression analysis for the alcoholic drink, HLA-DRB1*04:*11*:*01* and DRB1*04:*92* associated with the bacterial load (multibacillary). (DOCX)
