# Supplementary figures and images for: Alleles of HLA-DRB1*04 Associated with Pulmonary Tuberculosis in Amazon Brazilian Population
Source: PLoS One. 2016 Feb 22;11(2):e0147543. doi: 10.1371/journal.pone.0147543 (PMC4764689; doi:10.1371/journal.pone.0147543)

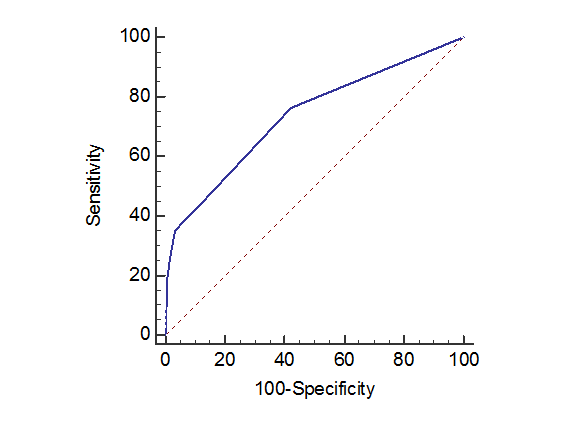

Supplement: S1 Fig — (TIF) [file pone.0147543.s001.tif]

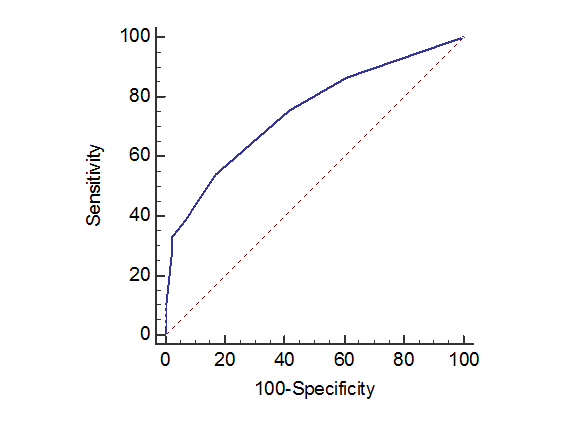

Supplement: S2 Fig — (TIF) [file pone.0147543.s002.tif]

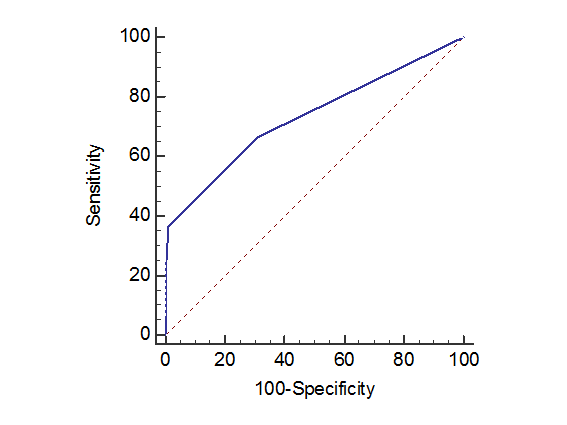

Supplement: S3 Fig — (TIF) [file pone.0147543.s003.tif]

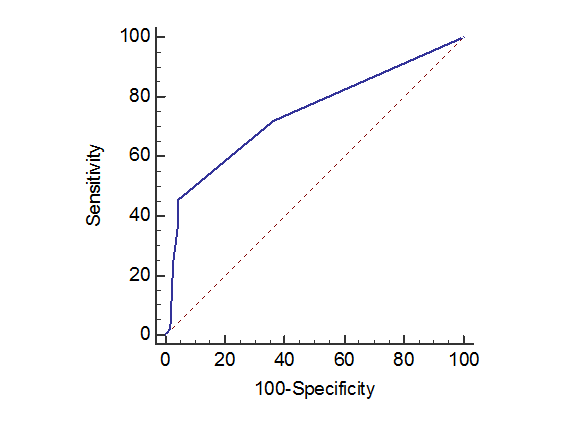

Supplement: S4 Fig — (TIF) [file pone.0147543.s004.tif]
