# Supplementary material for: Alleles of HLA-DRB1*04 Associated with Pulmonary Tuberculosis in Amazon Brazilian Population
Source: PLoS One. 2016 Feb 22;11(2):e0147543. doi: 10.1371/journal.pone.0147543 (PMC4764689; doi:10.1371/journal.pone.0147543)
Supplement: S1 Table — (DOCX) [file pone.0147543.s005.docx]

**S1 Table.** Stepwise logistic regression analysis with general characteristics of the population

| **Variables** | ***p* value** | **OR** | **95% CI** |
| --- | --- | --- | --- |
| Alcoholic drink | 0.0016 | 4.32 | 1.74 to 10.7 |
| Gender (Male) | 0.0001 | 2.69 | 1.64 to 4.40 |
| Smoking | 0.0442 | 3.16 | 1.03 to 9.69 |

Hosmer-Lemeshow test *p*=0.523; OR = Odds ratio; CI = Confidence interval.
