# Supplementary material for: Alleles of HLA-DRB1*04 Associated with Pulmonary Tuberculosis in Amazon Brazilian Population
Source: PLoS One. 2016 Feb 22;11(2):e0147543. doi: 10.1371/journal.pone.0147543 (PMC4764689; doi:10.1371/journal.pone.0147543)
Supplement: S2 Table — (DOCX) [file pone.0147543.s006.docx]

**S2 Table.** Stepwise logistic regression for variables alcoholic drink, gender (male) and smoking, including the *HLA-DRB1*04* gene.

| **Variables** | ***p* value** | **OR** | **95% CI** |
| --- | --- | --- | --- |
| Alcoholic drink | < 0.0001 | 8.43 | 3.85 to 18.5 |
| Gender (Male) | 0.0001 | 2.71 | 1.66 to 4.43 |
| Smoking | 0.0564 | 2.99 | 0.97 to 9.26 |
| *HLA-DRB1*04* | 0.0143 | 1.92 | 1.14 to 3.23 |

Hosmer-Lemeshow test *p*=0.260; OR = Odds ratio; CI = Confidence interval.
