# Supplementary material for: Alleles of HLA-DRB1*04 Associated with Pulmonary Tuberculosis in Amazon Brazilian Population
Source: PLoS One. 2016 Feb 22;11(2):e0147543. doi: 10.1371/journal.pone.0147543 (PMC4764689; doi:10.1371/journal.pone.0147543)
Supplement: S3 Table — (DOCX) [file pone.0147543.s007.docx]

**S3 Table.** Stepwise logistic regression analysis for alcohol and *HLA-DRB1*04:11:01* allele associated with pulmonary TB

| **Variables** | ***p* value** | **OR** | **95% CI** |
| --- | --- | --- | --- |
| Alcoholic drink | 0.0001 | 51.3 | 6.81 to 386 |
| *HLA-DRB1*04:11:01* | 0.0265 | 2.13 | 1.09 to 4.14 |

Hosmer-Lemeshow test *p*=0.287; OR = Odds ratio; CI = Confidence interval.
