# Supplementary material for: Alleles of HLA-DRB1*04 Associated with Pulmonary Tuberculosis in Amazon Brazilian Population
Source: PLoS One. 2016 Feb 22;11(2):e0147543. doi: 10.1371/journal.pone.0147543 (PMC4764689; doi:10.1371/journal.pone.0147543)
Supplement: S4 Table — (DOCX) [file pone.0147543.s008.docx]

**S4 Table.** Stepwise logistic regression analysis for the alcoholic drink, *HLA-DRB1*04:11:01* and *DRB1*04:92* associated with the bacterial load (multibacillary)

| **Variables** | ***p* value** | **OR** | **95% CI** |
| --- | --- | --- | --- |
| Alcoholic drink | 0.0026 | 11.1 | 3.99 to 30.9 |
| *HLA-DRB1*04:92* | 0.0112 | 8.62 | 1.63 to 45.5 |
| *HLA-DRB1*04:11:01* | 0.0442 | 2.01 | 1.03 to 3.93 |

Hosmer-Lemeshow test *p*=0.336; OR = Odds ratio; CI = Confidence interval.
